# Positive Selection Drives the Evolution of the Structural Maintenance of Chromosomes (SMC) Complexes

**DOI:** 10.3390/genes15091159

**Published:** 2024-09-03

**Authors:** Diego Forni, Alessandra Mozzi, Manuela Sironi, Rachele Cagliani

**Affiliations:** Computational Biology Unit, Scientific Institute IRCCS E. MEDEA, 23842 Bosisio Parini, Italy; diego.forni@lanostrafamiglia.it (D.F.); alessandra.mozzi@lanostrafamiglia.it (A.M.); manuela.sironi@lanostrafamiglia.it (M.S.)

**Keywords:** structural maintenance of chromosomes, meiotic cohesins, positive selection, host-pathogen arms race, intragenomic conflict

## Abstract

Structural Maintenance of Chromosomes (SMC) complexes are an evolutionary conserved protein family. In most eukaryotes, three SMC complexes have been characterized, as follows: cohesin, condensin, and SMC5/6 complexes. These complexes are involved in a plethora of functions, and defects in SMC genes can lead to an increased risk of chromosomal abnormalities, infertility, and cancer. To investigate the evolution of SMC complex genes in mammals, we analyzed their selective patterns in an extended phylogeny. Signals of positive selection were identified for condensin NCAPG, for two SMC5/6 complex genes (*SMC5* and *NSMCE4A*), and for all cohesin genes with almost exclusive meiotic expression (*RAD21L1*, *REC8*, *SMC1B*, and *STAG3*). For the latter, evolutionary rates correlate with expression during female meiosis, and most positively selected sites fall in intrinsically disordered regions (IDRs). Our results support growing evidence that IDRs are fast evolving, and that they most likely contribute to adaptation through modulation of phase separation. We suggest that the natural selection signals identified in SMC complexes may be the result of different selective pressures: a host-pathogen arms race in the condensin and SMC5/6 complexes, and an intragenomic conflict for meiotic cohesin genes that is similar to that described for centromeres and telomeres.

## 1. Introduction

Structural Maintenance of Chromosomes (SMC) complexes are an evolutionary conserved protein family present in many species, from bacteria to humans [1]. In most eukaryotes, three SMC complexes have been characterized, as follows: cohesin, condensin, and SMC5/6 complexes [1]. Such complexes are involved in a plethora of functions, including mitotic and meiotic chromosome condensation, sister chromatid cohesion, accurate chromosome segregation, DNA replication and repair, genome compartmentalization, and transcriptional regulation. All SMC complexes share structural features. Each complex is composed of three core proteins (two SMC proteins and a kleisin subunit) and peripheral subunits, forming a ring-shaped structure [1,2].

The cohesin complex is most likely the best studied SMC complex. In mammalian cells, the cohesin complex comprises two SMC proteins (SMC3 and SMC1A or SMC1B), an α-kleisin subunit (RAD21, RAD21L, or REC8), and a stromal antigen protein (STAG1, 2, or 3) [2]. Four of these subunits (REC8, RAD21L1, SMC1B, and STAG3) have an almost exclusive meiotic expression, and are therefore referred to as meiotic-specific cohesins. Hereafter, for the sake of simplicity, SMC3 (present in all cohesin complexes) and the remaining cohesin subunits (expressed preferentially in somatic cells) will be designated as non-meiotic cohesins (mitotic cohesins). Cohesin complexes are involved in a number of different mechanisms, from keeping sister chromatids together to contributing to the compartmentalization of chromosomes in topologically associative domains (TADs). Chromosome and nuclear compartmentalization, as well as TAD assembly, are mediated by phase separation. It has recently been reported that a fraction of cohesin associates with chromatin in a manner consistent with bridging-induced phase separation (BIPS, also known as polymer–polymer phase separation) [3,4]. BIPS uses multivalent protein–DNA interactions, bridging two distinct DNA regions and forming a DNA loop that acts as a nucleation structure for phase condensation [3,5]. In addition, during meiosis, meiotic-specific cohesins mediate Sister Chromatid Cohesion (SCC), the Synaptonemal Complex (SC) assembly and synapsis, as well as telomere attachment to the nuclear envelope, and telomere maintenance. The essential role of the cohesin complex in many aspects of chromosome biology is supported by the fact that defects in cohesin genes can lead to different diseases in which chromatid cohesion, DNA repair, transcriptional regulation, and genome topology are altered. Mutations in meiotic-specific cohesin genes have been associated with infertility, age-related aneuploidy, and premature ovarian failure [6]. Moreover, mutations in non-meiotic cohesin complex components and in their regulators have been associated with cancer [7,8,9]. Globally, mutations in these genes lead to disease conditions, also known as cohesinophaties. Among these, Cornelia de Lange syndrome (CdLS) is the most frequent and best-known disorder [10,11]. CdLS is a malformative syndrome in which intellectual and growth disorders are the main phenotypic manifestations [12,13]. Patients require lifelong rehabilitation, and about 80% of cases carry mutations in one of the cohesin complex components, or in one of their regulators (*SMC3*, *SMC1A*, *RAD21*, *STAG1*, *STAG2*, *HDAC8*, *NIPBL*) [11,12,14].

In addition to the cohesin complex, most eukaryotic genomes contain two distinct condensin complexes (Condensin I and II), which differ in their non-SMC subunits, in their cellular localization, and in their regulation during the cell cycle [15,16,17]. In particular, Condensin I localizes in the cytoplasm and gains access to chromosomes between prometaphase and telophase, when the nuclear envelope breaks down (NEBD). Conversely, Condensin II has a nuclear localization and, in mitosis, it binds stably to chromatin. Like cohesins, the condensin complex plays a key role in chromosome condensation, assembly, and segregation during mitosis and meiosis [18,19,20]. Condensins have also been associated with pathological conditions, as mutations in condensin subunits result in microcephaly due to impaired DNA decatenation [21,22].

The third member of SMC family, the SMC5/6 complex, has important functions in DNA repair by recombination, but also plays a role in influencing genome stability and dynamics in undamaged cells [23,24]. Furthermore, by preventing accumulation of toxic recombination intermediates, SMC5/6 promotes correct mitotic and meiotic chromosome segregation [23,24]. As in the case of cohesins, protein levels of SMC5/6 components decrease with age in mouse oocytes [25]. It was thus speculated that, in humans, reduced SMC5/6 availability may be associated with the increased risk of chromosomal abnormalities and infertility linked to maternal age. Moreover, mutations in *NSMCE2* or *NSMCE3* have been described in patients with primordial dwarfism, extreme insulin resistance, gonadal failure [26], and chromosome breakage syndrome and lung disease immunodeficiency [27]. Finally, the complex acts as a host-restriction factor, inhibiting the transcription of the genome of different viruses (i.e., HBV, unintegrated HIV1, HSV1, HCMV, KSHV, and HPV) [28,29,30,31,32,33,34,35,36].

Due to their essential functions and association with pathological conditions, SMC complex proteins would be expected to evolve under strict evolutionary constraint. Nevertheless, King and colleagues [37] recently observed signatures of recurrent positive selection in the Condensin II and in mitotic cohesin complexes across *Drosophila* and mammals. They also suggested the presence of an evolutionary arms race driven by viral infections.

To better understand the selective events underlying the evolution of genes that encode SMC complex proteins, we analyzed the selective patterns of the proteins that contribute to the formation of Cohesin, Condensin, and SMC5/6 complexes. 

## 2. Materials and Methods

### 2.1. Sequence Retrieval and Alignment

In this study we analyzed 26 subunits of cohesin, condensin I and II, and SMC5/6 complexes (Table 1), reported as “subunits” by Haering and Gruber [2]. Mammalian homologs of human genes were included only if they represented 1-to-1 orthologs, as reported in the EnsemblCompara GeneTrees [38]. Coding sequence information for at least 46 mammalian species was retrieved from the NCBI database (http://www.ncbi.nlm.nih.gov/, accessed on 15 July 2024), and from the UCSC server *(*http://genome.ucsc.edu/, accessed on 15 July 2024). The list of species and the number of sequences analyzed for each gene are reported in Table 1 and Supplementary Table S1.

The RevTrans 2.0 utility was used to generate Multiple Sequence Alignments (MSAs) using MAFFT as an aligner [39]. All alignments were manually inspected, and manual editing was used to correct misalignments in the proximity of small gaps. Phylogenetic trees were reconstructed using the phyML program with a General Time Reversible (GTR) model, γ-distributed rates, and 4 substitution rate categories with a fixed proportion of invariable sites [40].

Because recombination can generate false positive inferences of positive selection [41,42], MSAs were screened for the presence of recombination using GARD (Genetic Algorithm Recombination Detection) [43]. GARD is a genetic algorithm implemented in the HYPHY suite (version 2.2.4) [44], which uses phylogenetic incongruence among segments in the alignment to detect the best-fit number and location of recombination breakpoints. No significant breakpoint was detected.

### 2.2. Evolutionary Analysis in Mammals

To detect positive selection, we used the codon-based codeml program, implemented in the PAML (Phylogenetic Analysis by Maximum Likelihood) suite [45]. We applied different site (NSsite) models; specifically, we compared models of gene evolution that allow (NSsite models M2a and M8) or disallow (NSsite models M1a and M7) a class of codons to evolve with dN/dS >1. To assess statistical significance, twice the difference of the likelihood (ΔlnL) for the models (M1a vs. M2a and M7 vs. M8) was compared to a χ^2^ distribution (2 degrees of freedom for both comparisons). To assure reliability, different codon substitution models (F3x4 and F61) were used.

To identify specific sites subject to positive selection, we applied three different analyses, as follows: (i) the Bayes Empirical Bayes (BEB) analysis (with a cutoff ≥0.90) [46], (ii) the Fast Unbiased Bayesian AppRoximation (FUBAR) (with a cutoff ≥0.90) [47], and (iii) the Fixed Effects Likelihood (FEL) (with a *p*-value cutoff <0.1) [48].

To be conservative, and to limit false positives, only sites detected using at least two methods were considered as positive selection targets.

The average nonsynonymous substitution (dN)/synonymous substitution (dS) rate ratio, and the dN−dS parameter were calculated using the Single-Likelihood Ancestor Counting (SLAC) method [48]. Inputs were the MSAs and phyML trees (see Section 2.1).

FEL, FUBAR, and SLAC analyses were run locally through the HYPHY suite [44].

The codeml Free Ratio (FR) model was used to estimate different values of dN/dS on the branches of the phylogeny [49]. The FR model assumes different dN/dS for each lineage and is compared with a null model with one dN/dS for the entire phylogeny. Statistical significance is assessed by comparing twice the ΔlnL of the two models with a χ^2^ distribution with degrees of freedom equal to the difference in model parameters.

### 2.3. Correlation with Meiotic Gene Expression

Gene expression changes (fold-change) during female and male mouse meiosis were retrieved from previous works [50,51]. The correlation between dN/dS and fold-changes was evaluated using Kendall’s correlation, a non-parametric test based on ranks.

### 2.4. Prediction of Disordered Regions and Functional Motifs

In order to identified intrinsically disordered regions (IDRs), we used the Metapredict V2 tool [52,53], that applies a deep-learning algorithm based on a consensus score calculated from eight different disorder predictors [53]. Metapredict V2 was run using default parameters, and IDRs were defined as consecutive disordered stretches longer than 30 residues. Prediction of functional motifs and nuclear localization signals was performed using PROSITE (https://prosite.expasy.org/, accessed on 15 July 2024) [54] and NLStradamus software (http://www.moseslab.csb.utoronto.ca/NLStradamus/, accessed on 15 July 2024) [55], respectively.

To predict regions prone to undergo PS, we applied the ParSe method version 2.0 [56,57]. This tool defines PS-promoting regions by using sequence-based calculations of hydrophobicity, α-helix propensity, and a model of the polymer scaling exponent (νmodel). The applied model also includes the effects of interactions between amino acids (*U π* for π–π and cation–π interactions and *U q* for charge-based effects) trained on the saturation concentration associated with protein PS (*c_sat_*) [58,59].

## 3. Results

### 3.1. Evolutionary Analysis in Mammals: SMC Complexes Evolve at Different Rates

We first aimed to comprehensively analyze the selective pressure acting on mammalian genes that encode proteins of SMC complexes. In particular, we analyzed the evolutionary history of twenty-six SMC genes in at least forty-six mammalian species, as follows: eleven cohesins (four of them meiosis-specific), eight condensins, and seven SMC5/6 genes (Table 1, Supplementary Table S1) [2].

For coding genes, the strength of selection can be quantified by comparing the rate of dN with that of dS. We thus calculated the average dN/dS ratio using the SLAC method [48]. dN/dS values greater than 1 are consistent with positive (diversifying) selection, whereas ratios lower than 1 indicate purifying selection (selective constraint). The expected dN/dS under selective neutrality is 1.

**Figure 1:**
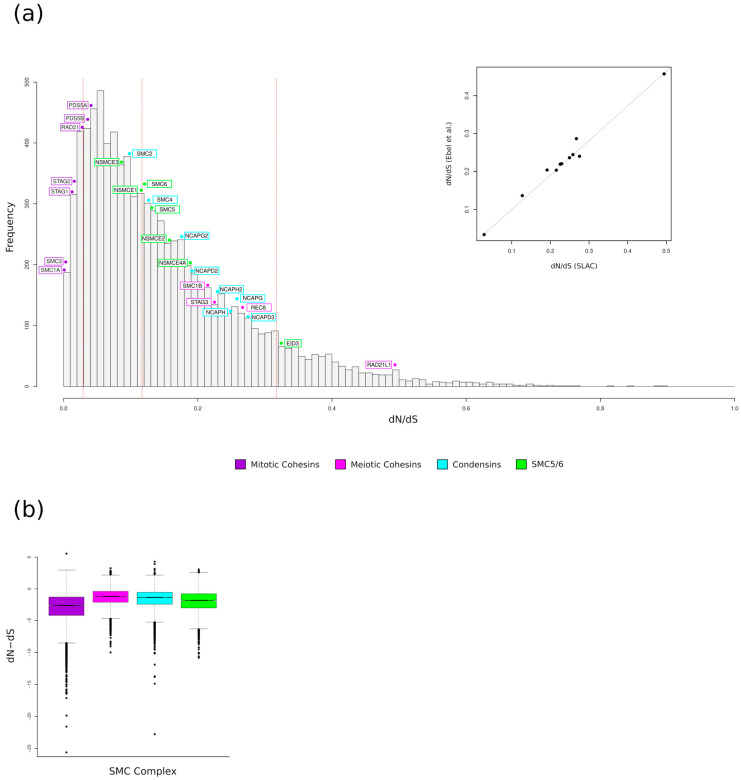
Evolutionary rates in SMC complexes. (**a**) Comparison of evolutionary rates. The distribution of dN/dS values for more than 9000 genes in a representative mammalian phylogeny [60] is shown. The hatched red lines correspond to the 10th, 50th, and 90th percentiles. The dN/dS values of the genes we analyzed are indicated. The inset shows the correlation between the dN/dS values we calculated and those previously reported by Ebel and coworkers for 11 SMC complex genes (*NCAPD2*, *NCAPD3*, *NCAPG*, *NCAPH*, *NCAPH2*, *RAD21*, *RAD21L*, *REC8*, *SMC1B*, *SMC4*, *STAG3*). (**b**) Boxplot representation of dN−dS values calculated for meiotic and mitotic Cohesin, Condensin, and SMC5/6 genes. Statistical significance was assessed by Nemenyi post hoc pairwise comparison after a Kruskal–Wallis test. All comparisons are significant, with a *p*-value < 0.001.

As reported in Table 1, all genes had dN/dS values much lower than 1, indicating that, as is the case for most mammalian genes [42], purifying selection is the major force acting on SMC complex genes. A comparison of dN/dS values among meiosis-specific and mitotic cohesin genes indicated that the latter tend to show a higher evolutionary constraint. The same results were observed by comparing mitotic cohesin genes with condensin and SMC5/6 genes (Table 1). To gain insight into the relative evolutionary rates of these proteins in a wider genomic context, we compared the average dN/dS values of the SMC complex genes to those previously calculated in more than 9000 genes in a representative mammalian phylogeny (24 species) [60]. In this phylogeny, the average dN/dS values were calculated for only eleven SMC genes, so we carried out a correlation analysis between dN/dS values obtained for these eleven genes on the two phylogenies (Figure 1a). There was a strong correlation (Spearman test, *p*-value = 2.2 × 10^−16^, rho = 0.95; Kendall test, *p*-value = 4.6 × 10^−5^, tau = 0.85) between dN/dS values calculated on our phylogeny and those calculated by Ebel and colleagues [60]; thus, we assumed we could compare our data to those calculated on a large gene set. As evident in Figure 1, all mitotic cohesin genes displayed the lowest dN/dS values among SMC genes, and their dN/dS values were well below the median for all human genes, confirming a stronger evolutionary constraint. Conversely, *RAD21L1* showed the fastest evolutionary rate among SMC complex genes, with a dN/dS value higher than the 98th percentile of the distribution.

To investigate the selection pattern of individual codons in SMC genes, we calculated the dN−dS parameter at each site [48]. dN−dS was preferred over the conventional dN/dS because it is not rendered to infinite for dS values equal to 0. The analysis was conducted on all the genes, that were categorized into the following four groups: meiotic and mitotic cohesins, condensin, and SMC5/6. All gene groups displayed a high proportion of constrained sites (dN−dS <0); in particular, mitotic cohesin genes were more constrained than the other gene groups, confirming the average dN/dS analysis. The distribution of dN−dS values was significantly different across the four gene groups (Figure 1b).

### 3.2. Positive Selection Drives the Evolution of Meiosis-Specific Cohesins

While constraints on protein function and structure typically result in overall purifying selection, diversifying selection is often limited to specific sites or domains [42]. Thus, to identify pervasive positive selection, we applied maximum likelihood analyses implemented in the PAML package [45,61]. In brief, we used the codeml program to compare models of gene evolution that allow (M2a and M8) or disallow (M1a and M7) a class of codons to evolve with dN/dS >1. The null models were rejected in favor of the positive selection models for all meiotic-specific cohesin genes (*RAD21L1*, *REC8*, *SMC1B*, and *STAG3*), for condensin *NCAPG*, and for two SMC5/6 complex genes (*SMC5* and *NSMCE4A*) (Table 2, Supplementary Tables S2–S4). Overall, these data indicate that a high proportion (27%) of SMC complex genes experienced positive selection. 

Previously, King and colleagues [37] reported signals of positive selection in all four mitotic cohesin genes analyzed (*SMC1*, *SMC3*, *RAD21*, and *STAG1*). This divergence between our results and those reported in the literature [37,62] may be due to several of the following factors. (i) In our analyses, we applied an extremely conservative approach; in fact, a gene was considered to be under positive selection only if all of the M1/M2 and M7/M8 comparisons for two codon frequency models (F3x4 and F61) were significant, while King and colleagues applied only one comparison (M7 vs. M8, model F3x4). (ii) The evolutionary analyses were conducted on different phylogenies; while our data derive from analyses carried out on an extended mammalian phylogeny, King and colleagues separately analyzed the different groups of mammals (primates, *Murinae*, *Cricetidae*, bats, and *Bovidae*).

To assess whether substitution saturation was responsible for the high fraction of positively selected genes we identified, we used the PAML Free Ratio (FR) model to estimate the dS for all branches of the gene phylogenies [49]. In the positively selected genes, a minority of branches had dS > 0.25, and very few had dS > 0.5 (Supplementary Table S5); this excludes the fact that dS saturation has a major effect on evolutionary inference.

Next, we sought to analyze selection patterns across the whole mammalian phylogeny. To this aim, we again applied the FR model. The FR model fit the data better than the null model for 21 genes (Supplementary Table S6), suggesting that, for these genes, the selective pressure has been acting differently across the phylogeny. To display specific lineages that carry natural selection signals, we overlaid the proportion of genes showing dN/dS > 1 over the mammalian tree for each of the three SMC complexes separately. Most of the branches leading to superorders/orders showed at least one gene with dN/dS > 1, for all SMC complex genes (Supplementary Figure S1). In particular, the branches leading to primates and laurasiatheria showed a relatively high number of selected genes. Similarly, for tip branches, selection appeared to be strong in primates. In general, weak selection signals were detected in rodents (Supplementary Figure S1).

### 3.3. Analysis of Positively Selected Sites

**Figure 2:**
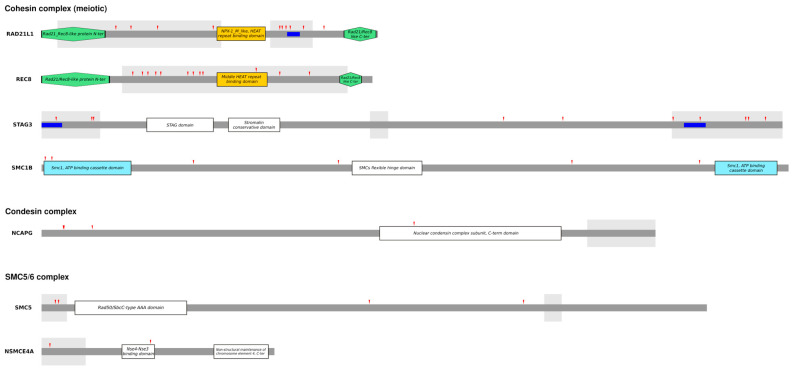
Domain structures of SMC complexes. Schematic domain structures of the 7 proteins with evidence of positive selection are drawn to scale. Domains are defined using the InterPro (https://www.ebi.ac.uk/interpro/, accessed on 15 July 2024) classification. The gray-shaded areas represent IDRs identified by the Metapredict tool based on human proteins. The red arrows denote positively selected sites as obtained from positive selection analysis. ParSe sequences are represented in blue.

To identify specific codons targeted by positive selection, we applied a conservative strategy. Specifically, we called a site positively selected only if it was detected by at least two of the following methods: BEB, FUBAR, or FEL (see Materials and Methods). The positively selected sites are reported in Table 2. Briefly, we identified forty-eight positively selected sites, thirty-eight of which are in meiotic cohesins (ten in RAD21L and STAG3, twelve in REC8, and six in SMC1B), four in NCAPG, four in SMC5, and two in NSMCE4A. Next, we aimed to investigate the potential functional effects of positive selection. By looking at the positions of the positively selected sites within the proteins, we observe that ~67% of sites are located in intrinsically disordered regions (IDRs) (Figure 2). IDRs are regions that do not adopt a stable three-dimensional structure, but rather exist in a collection of structurally distinct conformers. Nevertheless, IDRs are known to play different regulatory functions in the cell, and to mediate protein–protein interactions, because their lack of structural constraints allows them to adapt their conformation to different interacting partners [63]. We thus tested whether in these genes IDRs are significantly enriched of positively selected sites. We found this to be the case for *RAD21L1*, *REC8*, *STAG3,* and *SMC5* (binomial test; *RAD21L1 p*-value: 0.01; *REC8 p*-value: 0.013; *STAG3 p*-value: 1.77 × 10^−4^; *SMC5 p*-value: 0.023). Moreover, proteins containing IDRs are known to be essential for phase separation (PS). PS plays a role in many biological processes, including chromosome organization [64,65,66]. We thus applied the ParSe (Partition Sequence) method [57] to identify regions that promote PS in the selected genes. PS-promoting regions were detected in the IDRs of RAD21L1 and STAG3. Interestingly, all three identified PS-promoting regions carry at least one positively selected site (Figure 2).

We then scanned protein sequences using the PROSITE tool to infer functional motifs. In summary, thirteen out of forty-eight positively selected sites fall in a functional motif, of which nine are phosphorylation sites, three are myristoylation sites, and one is a glycosylation site. Notably, in NCAPG, three out of four positively selected sites are phosphorylation sites, recognized by Protein Kinase C (site thirty-six) and Casein Kinase 2 (sites thirty-seven and eighty-four), of which site thirty-seven involves the residue that is phosphorylated. Finally, since most SMC complex components have nuclear expression, we looked for nuclear localization signals in positively selected genes. In STAG3, two positively selected sites (R83 and H86) fall under nuclear localization signals predicted by the NLStradamus software.

### 3.4. Meiotic Cohesin Evolutionary Rates Correlate with Expression during Female Meiosis

**Figure 3:**
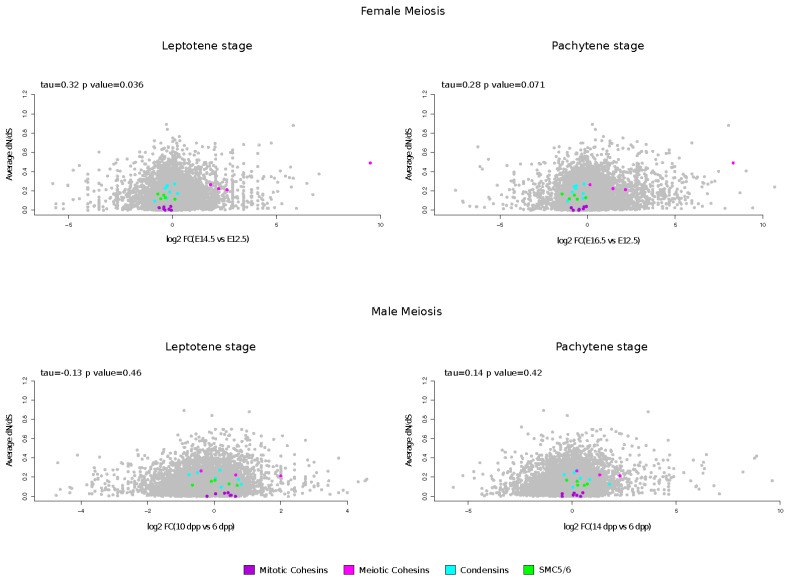
Evolutionary rates and gene expression in meiosis. Average dN/dS for all SMC complex genes is plotted against the log2 fold-change (FC) of gene expression in the leptotene or pachytene stages vs. the pre-meiotic stage of mouse oogenesis or spermatogenesis. Kendall’s correlation coefficients are also reported.

As reported above, meiosis-specific Cohesin genes displayed a high average of dN/dS values, and were found to be positively selected. Thus, we investigated the relationship between evolutionary rates and gene meiotic expression, using genome-wide RNA-seq data for fetal mouse ovaries to recover information on gene expression before and during meiosis [50]. In particular, we obtained expression level changes (fold-change) for the leptotene (E14.5) and pachytene (E16.5) stages compared to a pre-meiotic (E12.5) stage. Furthermore, we retrieved expression changes during different stages of male mouse meiosis compared to pre-meiotic stages (6 days post-partum, dpp). Specifically, time periods that roughly correspond to the leptotene/zygotene stage and the pachytene stage (10 dpp and 14 dpp, respectively) were analyzed [51]. Finally, these values were correlated to the average dN/dS. A positive correlation was obtained for the leptotene stage of female meiosis, whereas a correlation with borderline significance was observed for the pachytene stage (Figure 3). Conversely, no significant correlation was observed between dN/dS and increased meiotic expression for male meiosis. As shown in the Figure 3, meiotic cohesin genes that are upregulated in female meiosis evolve faster than mitotic cohesins; in the latter, condensin and SMC5/6 subunits show no or limited upregulation during meiosis.

## 4. Discussion

Chromosomal DNA rearrangements drive and facilitate diverse genomic processes, including chromosome segregation, gene expression, DNA repair, and recombination. SMC complexes are involved in these fundamental processes of genome organization; they are essential for all organisms across the tree of life, and they are deeply conserved in eukaryotes [1]. The importance of these complexes is not limited to mitosis and meiosis, where, in fact, they are fundamental, but they participate with different functions throughout the entire cell cycle [16]. The pivotal role played by the SMC components is confirmed by two other pieces of evidence, as follows: (i) mutations in SMC genes determine pathological conditions, including tumor forms; (ii) some of these genes are targets of natural selection, as previously reported in *Drosophila*, and in some mammalian groups [37,62]. In these studies, evolutionary analyses have only been conducted on a limited number of SMC genes. Thus, we aimed to cover this gap by analyzing the evolutionary history of all of the components of the SMC complexes, including meiotic cohesins, which were never analyzed previously. Indeed, given the key role of these genes in the regulation of primary biological processes of the cell machinery, many different selective forces are expected to drive their evolution.

Our observations on the genes of the cohesin complexes are particularly interesting. In these genes, two distinct trends are highlighted. On one hand, the mitotic cohesins are highly constrained; on the other hand, the meiotic cohesins show signals of pervasive positive selection. In all cohesin genes with predominantly meiotic expression, we identified strong positive selection signals and found that the selected sites are significantly clustered within IDRs, supporting growing evidence that IDRs are fast evolving in different systems [67,68,69,70,71,72,73]. Proteins containing IDRs are known to be essential for phase separation (PS), a process that consists of the compartmentalization of proteins and nucleic acids within the cell, and plays a role in a wide range of processes, such as chromosome dynamics, meiotic chromosome organization, and meiotic sex chromosome inactivation (MSCI) [64,65,66,74]. A series of meiosis-specific events, including programmed DNA double-strand break formation, homologous pairing, synaptonemal complex installation, and inter-homolog crossover formation, take place to ensure successful chromosome segregation. During meiosis, cohesins and chromosomal phase separation are fundamental in these processes. In this light, we suggest that meiotic cohesins may be engaged in an intragenomic conflict similar to the ones previously described for centromeres, telomeres, and telomere/centromere-binding proteins [75,76,77]. The centromere drive hypothesis postulates that selfish centromeric DNA elements favor their preferential inclusion in the oocyte through the recruitment of kinetochore components. Similarly, we previously proposed that selfish subtelomeric DNA elements can influence the directionality of chromosome movements to the centrosome during meiosis, and that this skews their segregation; the fast evolution of telomere-binding proteins would thus serve the purpose of suppressing meiotic drive, and would restore equal partitioning [75]. Because cohesins can potentially influence chromosome movement during meiosis, they may also participate in the control of cheating DNA elements to ensure proper segregation. In support of this hypothesis, we detected a significant correlation between the evolutionary rate of meiotic cohesin genes and their upregulation during female mouse meiosis. We thus suggest that cohesins join centromere- and telomere-binding proteins as elements involved in intragenomic conflicts fueled by selfish elements that promote meiotic drive. Furthermore, MSCI is considered a driving force for genomic evolution. In particular, germline X chromosome inactivation, which occurs in the germ cells of XY males, has been linked to genetic conflicts related to sexual antagonism [78]. Thus, an alternative, non-mutually exclusive possibility is that meiotic cohesins are involved in an intragenetic conflict related to MSCI.

The SMC5/6 complex, in addition to its physiological roles in chromosome maintenance (repair of chromosomal DNA, conformational compaction of bound DNA, DNA replication), functions as a host restriction factor against several viruses, including HBV, unintegrated HIV-1, papillomavirus (HPV), and different herpesviruses (KSHV, EBV, HSV-1) [30]. The SMC5/6 complex recognizes and binds viral episomal DNA molecules, inducing their epigenetic silencing. In turn, episomal DNA viruses antagonize the function of the SMC5/6 complex by expressing viral proteins that degrade one or more of the SMC5/6 components. For example, the HBV HBx protein recruits cellular DDB1 (DNA damage-binding protein 1), with an E3 ubiquitin ligase that targets SMC5/6 for proteasomal degradation. This antagonism of the SMC5/6 complex by HBx is an evolutionarily conserved function found in divergent mammalian HBV species [62], and it leads to the specific degradation of SMC5 and SMC6 components [28,29]. A similar function is reported for EBV BNRF1 and KSHV RTA [34,36].

In general, these observations suggest that components of the SMC5/6 complex are engaged in a host-pathogen genetic conflict. The latter ensues when a host restriction factor targets one or more viruses, which evolve counter-restriction mechanisms. The viral proteins mutate to escape restriction by the host factor, which in turn evolves to re-establish viral restriction. This cycle recurs repeatedly and results in an evolutionary arms race [79].

The arms race with viral pathogens may underlie the positive selection signal identified in the following two components of the SMC5/6 complex, as both are directly involved in the pathogen-host conflict: SMC5 is an HBV Hbx target for proteasomal degradation, while NSMCE4A interacts with episomal DNA template.

Mammals have two Nse4 paralogs encoded by *NSMCE4A* and *EID3.* NSE4a and NSE4b share two highly conserved kleisin domains, and are equally efficient at supporting the assembly of a full SMC5/6 complex. Nevertheless, it has been suggested that SMC5/6 containing NSE4a or NSE4b may exhibit different DNA-binding substrate preferences [80]. Indeed, the Nse4a-containing SMC5/6 complex exhibits episomal restriction activity, and has been recovered in HBx pull-down experiments. In contrast, the Nse4b-containing SMC5/6 complex is defective in its interaction with the episomal DNA template, supporting our hypothesis that the positive selection signals identified in the *NSMCE4A* gene (but not in the *EID3* gene) arise from a host-pathogen conflict.

An evolutionary conflict between hosts and pathogens could also underlie the positive selection found in NCAPG. By acting on the condensin complex, gammaherpesviruses are able to induce host chromosomal condensation to promote the replication of the viral genome. EBV is known to activate the condensin complex by NCAPG phosphorylation [81]. Specifically the viral BGLF4 kinase induces NCAPG phosphorylation at the Cdc2 target motifs, suggesting that the viral kinase might induce chromosome condensation by mimicking Cdc2. The Condensin I complex is constitutively present all throughout the cell cycle, and it regulates the state of chromatin condensation, which is in a relaxed form during interphase, and is converted into compact rod-like structures during mitosis. The function of Condensin I must be tightly regulated during the cell cycle, and this occurs through the phosphorylation of its components by different kinases. Three of the four positively selected sites in NCAPG fall into phosphorylation sites, and in particular, site thirty-seven corresponds to the residue that is phosphorylated by Casein Kinase 2 (CK2). CK2 is the main kinase that phosphorylates Condensin I during interphase and reduces its supercoiling activity, in contrast to the slight stimulatory effect of mitosis-specific phosphorylation by Cdc2 [82]. We speculate that NCAPG phosphorylation sites other than Cdc2 sites may be the targets of viral kinases, determining the effects of natural selection on this gene, and in particular on CK2 phosphorylation sites.

In conclusion, by analyzing a mammalian phylogeny that spans ~99 million years of evolution [83], we show that the natural selection signals identified in SMC complexes may be the result of different selective pressures. Regarding the selection signals in the condensin and SMC5/6 complexes, the data suggest a host-pathogen arms race. In contrast, the evolutionary rate of meiotic cohesin genes could be the result of an intragenomic conflict similar to that described for centromeres and telomeres.

## Figures and Tables

**Table 1:** List of analyzed SMC complex genes.

	Gene	Alias Gene Symbol	Subunits	n. of Species	dN/dS
Cohesin complex					
	RAD21	SCC1	Kleisin	63	0.028
	RAD21L1*	RAD21L	Kleisin	63	0.494
	REC8*	-	Kleisin	63	0.267
	SMC1A	-	SMC	61	0.003
	SMC1B*	-	SMC	60	0.215
	SMC3	-	SMC	63	0.001
	PDS5A	SCC112	HEAT-A	57	0.041
	PDS5B	APRIN, AS3	HEAT-A	60	0.036
	STAG1	SA1	HEAT-B	63	0.013
	STAG2	SA2	HEAT-B	63	0.016
	STAG3*	SA3	HEAT-B	63	0.225
Condensin complex					
	NCAPD2	CAP-D2	HEAT-A (I)	62	0.191
	NCAPD3	CAP-D3	HEAT-A (II)	63	0.275
	NCAPG	CAP-G	HEAT-B (I)	63	0.258
	NCAPG2	CAP-G2	HEAT-B (II)	59	0.176
	NCAPH	CAP-H	Kleisin (I)	63	0.249
	NCAPH2	CAP-H2	Kleisin (II)	62	0.229
	SMC2	CAP-E	SMC	62	0.098
	SMC4	CAP-C	SMC	61	0.127
SMC5/6 complex					
	NSMCE1	NSE1	Tandem-WHD E3 ligase	60	0.120
	NSMCE2	NSE2	SUMO ligase	63	0.158
	NSMCE3	NSE3/MAGEG1	Tandem-WHD	54	0.087
	NSMCE4A	NSE4A	Kleisin	63	0.189
	EID3	NSMCE4B	Kleisin	46	0.342
	SMC5	-	SMC	63	0.131
	SMC6	-	SMC	63	0.116

**Table 2:** Likelihood ratio test statistics for models of variable selective pressure among sites (F3x4 and F61 codon frequency model) for SMC complexes.

	Gene/LRT Model	n. of Species	F3x4		F61		Positively Selected Sites ^c^
			−2ΔlnL ^a^	*p*-value ^b^	−2ΔlnL ^a^	*p*-value ^b^	
Cohesin Complex							
	RAD21L*	63					
	M1 vs. M2		102.59	5.28 × 10^−23^	92.78	7.13 × 10^−21^	122, 148, 192, 284, 394, 398, 404, 411, 477, 433
	M7 vs. M8		113.97	1.79 × 10^−25^	108.91	2.25 × 10^−24^	
	REC8*	63					
	M1 vs. M2		51.13	7.89 × 10^−12^	10.11	0.0064	152, 168, 191, 199, 253, 264, 269, 358, 400, 449, 178, 244
	M7 vs. M8		88.22	6.97 × 10^−20^	50.28	1.21 × 10^−11^	
	SMC1B*	60					
	M1 vs. M2		37.77	6.29 × 10^−9^	16.92	0.00021	6, 18, 251, 491, 877, 1088
	M7 vs. M8		105.04	1.55 × 10^−23^	55.29	9.85 × 10^−13^	
	STAG3*	62					
	M1 vs. M2		27.39	1.13 × 10^−6^	18.02	0.00012	24, 83, 86, 764, 862, 1044, 1089, 1154, 1159, 1197
	M7 vs. M8		79.88	4.51 × 10^−18^	58.44	2.04 × 10^−13^	
Condensin Complex							
	NCAPG	63					
	M1 vs. M2		46.98	6.29 × 10^−11^	48.72	2.63 × 10^−11^	36, 37, 84, 616
	M7 vs. M8		90.97	1.76 × 10^−20^	102.35	5.96 × 10^−23^	
SMC5/6 Complex							
	SMC5	63					
	M1 vs. M2		17.97	0.000125	7.91	0.019	33, 38,542, 797
	M7 vs. M8		61.40	4.65 × 10^−14^	45.29	1.46 × 10^−10^	
	NSMCE4A	63					
	M1 vs. M2		33.96	4.22 × 10^−8^	22.82	1.11 × 10^−5^	14, 185
	M7 vs. M8		45.11	1.60 × 10^−10^	35.79	1.69 × 10^−8^	

^a^ Twice the difference of likelihood for the two models compared; ^b^ *p*-value of rejecting the neutral models (M8a and M7) in favor of the positive selection model (M8); ^c^ positively selected sites detected by at least two methods among BEB, FEL, and FUBAR.

## Data Availability

The data that support the findings of this study are included in the article; further inquiries can be directed to the corresponding author upon reasonable request.

## Supplementary Materials

The following supporting information can be downloaded at: https://www.mdpi.com/article/10.3390/genes15091159/s1, Supplementary Figure S1. dN/dS variability among mammalian species. Representative phylogenetic tree of mammalian species analyzed herein. Colored dots represent tree branches showing dN/dS value >1 estimated by the MFR model for that specific group. See the legend for color details, Supplementary Table S1. List of species analyzed in each SMC gene. A white square indicates that the species is missing for that gene, Supplementary Table S2. Likelihood ratio test statistics for models of variable selective pressure among sites (F3x4 and F61 codon frequency model) for Cohesin Complex genes, Supplementary Table S3. Likelihood ratio test statistics for models of variable selective pressure among sites (F3x4 and F61 codon frequency model) for Condensin Complex genes, Supplementary Table S4. Likelihood ratio test statistics for models of variable selective pressure among sites (F3x4 and F61 codon frequency model) for SMC5/6 complex genes, Supplementary Table S5. Analysis of dS for individual branches in the phylogenies of positively selected genes, Supplementary Table S6. Likelihood ratio test statistics for models of variable selective pressure among branches.

## References

[1] Uhlmann F. (2016). SMC Complexes: From DNA to Chromosomes. Nat. Rev. Mol. Cell Biol..

[2] Haering C.H., Gruber S. (2016). SnapShot: SMC Protein Complexes Part I. Cell.

[3] Park J., Kim J.-J., Ryu J.-K. (2024). Mechanism of Phase Condensation for Chromosome Architecture and Function. Exp. Mol. Med..

[4] Ryu J.-K., Bouchoux C., Liu H.W., Kim E., Minamino M., De Groot R., Katan A.J., Bonato A., Marenduzzo D., Michieletto D. (2021). Bridging-Induced Phase Separation Induced by Cohesin SMC Protein Complexes. Sci. Adv..

[5] Erdel F., Rippe K. (2018). Formation of Chromatin Subcompartments by Phase Separation. Biophys. J..

[6] Beverley R., Snook M.L., Brieño-Enríquez M.A. (2021). Meiotic Cohesin and Variants Associated with Human Reproductive Aging and Disease. Front. Cell Dev. Biol..

[7] Hill V.K., Kim J.-S., Waldman T. (2016). Cohesin Mutations in Human Cancer. Biochim. Biophys. Acta (BBA) Rev. Cancer.

[8] Pati D. (2024). Role of Chromosomal Cohesion and Separation in Aneuploidy and Tumorigenesis. Cell. Mol. Life Sci..

[9] Di Nardo M., Pallotta M.M., Musio A. (2022). The Multifaceted Roles of Cohesin in Cancer. J. Exp. Clin. Cancer Res..

[10] Liu J., Krantz I.D. (2009). Cornelia de Lange Syndrome, Cohesin, and Beyond. Clin. Genet..

[11] Barbero J.L. (2013). Genetic Basis of Cohesinopathies. Appl. Clin. Genet..

[12] Kline A.D., Moss J.F., Selicorni A., Bisgaard A.-M., Deardorff M.A., Gillett P.M., Ishman S.L., Kerr L.M., Levin A.V., Mulder P.A. (2018). Diagnosis and Management of Cornelia de Lange Syndrome: First International Consensus Statement. Nat. Rev. Genet..

[13] Kline A.D., Krantz I.D., Sommer A., Kliewer M., Jackson L.G., FitzPatrick D.R., Levin A.V., Selicorni A. (2007). Cornelia de Lange Syndrome: Clinical Review, Diagnostic and Scoring Systems, and Anticipatory Guidance. Am. J. Med. Genet. Part A.

[14] Yuan B., Neira J., Pehlivan D., Santiago-Sim T., Song X., Rosenfeld J., Posey J.E., Patel V., Jin W., Adam M.P. (2019). Clinical Exome Sequencing Reveals Locus Heterogeneity and Phenotypic Variability of Cohesinopathies. Genet. Med..

[15] Hirano T. (2016). Condensin-Based Chromosome Organization from Bacteria to Vertebrates. Cell.

[16] Hoencamp C., Rowland B.D. (2023). Genome Control by SMC Complexes. Nat. Rev. Mol. Cell. Biol..

[17] Ono T., Fang Y., Spector D.L., Hirano T. (2004). Spatial and Temporal Regulation of Condensins I and II in Mitotic Chromosome Assembly in Human Cells. Mol. Biol. Cell.

[18] Cuylen S., Haering C.H. (2011). Deciphering Condensin Action during Chromosome Segregation. Trends Cell Biol..

[19] Kinoshita K., Hirano T. (2017). Dynamic Organization of Mitotic Chromosomes. Curr. Opin. Cell Biol..

[20] Kakui Y., Uhlmann F. (2018). SMC Complexes Orchestrate the Mitotic Chromatin Interaction Landscape. Curr. Genet..

[21] Martin C.-A., Murray J.E., Carroll P., Leitch A., Mackenzie K.J., Halachev M., Fetit A.E., Keith C., Bicknell L.S., Fluteau A. (2016). Mutations in Genes Encoding Condensin Complex Proteins Cause Microcephaly through Decatenation Failure at Mitosis. Genes Dev..

[22] Pang D., Yu S., Yang X. (2022). A Mini-Review of the Role of Condensin in Human Nervous System Diseases. Front. Mol. Neurosci..

[23] Peng X.P., Zhao X. (2023). The Multi-Functional Smc5/6 Complex in Genome Protection and Disease. Nat. Struct. Mol. Biol..

[24] Aragón L. (2018). The Smc5/6 Complex: New and Old Functions of the Enigmatic Long-Distance Relative. Annu. Rev. Genet..

[25] Hwang G., Sun F., O’Brien M., Eppig J.J., Handel M.A., Jordan P.W. (2017). SMC5/6 Is Required for the Formation of Segregation-Competent Bivalent Chromosomes during Meiosis I in Mouse Oocytes. Development.

[26] Payne F., Colnaghi R., Rocha N., Seth A., Harris J., Carpenter G., Bottomley W.E., Wheeler E., Wong S., Saudek V. (2014). Hypomorphism in Human NSMCE2 Linked to Primordial Dwarfism and Insulin Resistance. J. Clin. Investig..

[27] Van Der Crabben S.N., Hennus M.P., McGregor G.A., Ritter D.I., Nagamani S.C.S., Wells O.S., Harakalova M., Chinn I.K., Alt A., Vondrova L. (2016). Destabilized SMC5/6 Complex Leads to Chromosome Breakage Syndrome with Severe Lung Disease. J. Clin. Investig..

[28] Decorsière A., Mueller H., Van Breugel P.C., Abdul F., Gerossier L., Beran R.K., Livingston C.M., Niu C., Fletcher S.P., Hantz O. (2016). Hepatitis B Virus X Protein Identifies the Smc5/6 Complex as a Host Restriction Factor. Nature.

[29] Murphy C.M., Xu Y., Li F., Nio K., Reszka-Blanco N., Li X., Wu Y., Yu Y., Xiong Y., Su L. (2016). Hepatitis B Virus X Protein Promotes Degradation of SMC5/6 to Enhance HBV Replication. Cell Rep..

[30] Irwan I.D., Cullen B.R. (2023). The SMC5/6 Complex: An Emerging Antiviral Restriction Factor That Can Silence Episomal DNA. PLoS Pathog..

[31] Xu W., Ma C., Zhang Q., Zhao R., Hu D., Zhang X., Chen J., Liu F., Wu K., Liu Y. (2018). PJA1 Coordinates with the SMC5/6 Complex to Restrict DNA Viruses and Episomal Genes in an Interferon-Independent Manner. J. Virol..

[32] Gibson R.T., Androphy E.J. (2020). The SMC5/6 Complex Represses the Replicative Program of High-Risk Human Papillomavirus Type 31. Pathogens.

[33] Bentley P., Tan M.J.A., McBride A.A., White E.A., Howley P.M. (2018). The SMC5/6 Complex Interacts with the Papillomavirus E2 Protein and Influences Maintenance of Viral Episomal DNA. J. Virol..

[34] Yiu S.P.T., Guo R., Zerbe C., Weekes M.P., Gewurz B.E. (2022). Epstein-Barr Virus BNRF1 Destabilizes SMC5/6 Cohesin Complexes to Evade Its Restriction of Replication Compartments. Cell Rep..

[35] Dupont L., Bloor S., Williamson J.C., Cuesta S.M., Shah R., Teixeira-Silva A., Naamati A., Greenwood E.J.D., Sarafianos S.G., Matheson N.J. (2021). The SMC5/6 Complex Compacts and Silences Unintegrated HIV-1 DNA and Is Antagonized by Vpr. Cell Host Microbe.

[36] Han C., Zhang D., Gui C., Huang L., Chang S., Dong L., Bai L., Wu S., Lan K. (2022). KSHV RTA Antagonizes SMC5/6 Complex-Induced Viral Chromatin Compaction by Hijacking the Ubiquitin-Proteasome System. PLoS Pathog..

[37] King T.D., Leonard C.J., Cooper J.C., Nguyen S., Joyce E.F., Phadnis N. (2019). Recurrent Losses and Rapid Evolution of the Condensin II Complex in Insects. Mol. Biol. Evol..

[38] Vilella A.J., Severin J., Ureta-Vidal A., Heng L., Durbin R., Birney E. (2009). EnsemblCompara GeneTrees: Complete, Duplication-Aware Phylogenetic Trees in Vertebrates. Genome Res..

[39] Wernersson R. (2003). RevTrans: Multiple Alignment of Coding DNA from Aligned Amino Acid Sequences. Nucleic Acids Res..

[40] Guindon S., Delsuc F., Dufayard J.F., Gascuel O. (2009). Estimating Maximum Likelihood Phylogenies with PhyML. Methods Mol. Biol..

[41] Anisimova M., Nielsen R., Yang Z. (2003). Effect of Recombination on the Accuracy of the Likelihood Method for Detecting Positive Selection at Amino Acid Sites. Genetics.

[42] Sironi M., Cagliani R., Forni D., Clerici M. (2015). Evolutionary Insights into Host-Pathogen Interactions from Mammalian Sequence Data. Nat. Rev. Genet..

[43] Pond S.L.K., Posada D., Gravenor M.B., Woelk C.H., Frost S.D. (2006). Automated Phylogenetic Detection of Recombination Using a Genetic Algorithm. Mol. Biol. Evol..

[44] Pond S.L.K., Frost S.D.W., Muse S.V. (2005). HyPhy: Hypothesis Testing Using Phylogenies. Bioinformatics.

[45] Yang Z. (2007). PAML 4: Phylogenetic Analysis by Maximum Likelihood. Mol. Biol. Evol..

[46] Anisimova M., Bielawski J.P., Yang Z. (2002). Accuracy and Power of Bayes Prediction of Amino Acid Sites Under Positive Selection. Mol. Biol. Evol..

[47] Murrell B., Wertheim J.O., Moola S., Weighill T., Scheffler K., Pond S.L.K. (2012). Detecting Individual Sites Subject to Episodic Diversifying Selection. PLoS Genet..

[48] Kosakovsky Pond S.L., Frost S.D.W. (2005). Not So Different After All: A Comparison of Methods for Detecting Amino Acid Sites Under Selection. Mol. Biol. Evol..

[49] Yang Z., Nielsen R. (1998). Synonymous and Nonsynonymous Rate Variation in Nuclear Genes of Mammals. J. Mol. Evol..

[50] Soh Y.Q., Junker J.P., Gill M.E., Mueller J.L., van Oudenaarden A., Page D.C. (2015). A Gene Regulatory Program for Meiotic Prophase in the Fetal Ovary. PLoS Genet..

[51] Margolin G., Khil P.P., Kim J., Bellani M.A., Camerini-Otero R.D. (2014). Integrated Transcriptome Analysis of Mouse Spermatogenesis. BMC Genom..

[52] .Emenecker R.J., Griffith D., Holehouse A.S. Metapredict V2: An update to metapredict, a fast, accurate, and easy-to-use predictor of consensus disorder and structure. bioRxiv..

[53] Emenecker R.J., Griffith D., Holehouse A.S. (2021). Metapredict: A Fast, Accurate, and Easy-to-Use Predictor of Consensus Disorder and Structure. Biophys. J..

[54] Sigrist C.J.A., De Castro E., Cerutti L., Cuche B.A., Hulo N., Bridge A., Bougueleret L., Xenarios I. (2012). New and Continuing Developments at PROSITE. Nucleic Acids Res..

[55] Nguyen Ba A.N., Pogoutse A., Provart N., Moses A.M. (2009). NLStradamus: A Simple Hidden Markov Model for Nuclear Localization Signal Prediction. BMC Bioinform..

[56] Ibrahim A.Y., Khaodeuanepheng N.P., Amarasekara D.L., Correia J.J., Lewis K.A., Fitzkee N.C., Hough L.E., Whitten S.T. (2023). Intrinsically Disordered Regions That Drive Phase Separation Form a Robustly Distinct Protein Class. J. Biol. Chem..

[57] Wilson C., Lewis K.A., Fitzkee N.C., Hough L.E., Whitten S.T. (2023). ParSe 2.0: A Web Tool to Identify Drivers of Protein Phase Separation at the Proteome Level. Protein Sci..

[58] Tesei G., Trolle A.I., Jonsson N., Betz J., Pesce F., Johansson K.E., Lindorff-Larsen K. (2023). Conformational Ensembles of the Human Intrinsically Disordered Proteome: Bridging Chain Compaction with Function and Sequence Conservation. bioRxiv.

[59] Tesei G., Lindorff-Larsen K. (2023). Improved Predictions of Phase Behaviour of Intrinsically Disordered Proteins by Tuning the Interaction Range. Open Res. Eur..

[60] Ebel E.R., Telis N., Venkataram S., Petrov D.A., Enard D. (2017). High Rate of Adaptation of Mammalian Proteins That Interact with Plasmodium and Related Parasites. PLoS Genet..

[61] Yang Z. (1997). PAML: A Program Package for Phylogenetic Analysis by Maximum Likelihood. Comput. Appl. Biosci. CABIOS.

[62] Abdul F., Filleton F., Gerossier L., Paturel A., Hall J., Strubin M., Etienne L. (2018). Smc5/6 Antagonism by HBx Is an Evolutionarily Conserved Function of Hepatitis B Virus Infection in Mammals. J. Virol..

[63] Wright P.E., Dyson H.J. (2015). Intrinsically Disordered Proteins in Cellular Signalling and Regulation. Nat. Rev. Mol. Cell. Biol..

[64] Strom A.R., Emelyanov A.V., Mir M., Fyodorov D.V., Darzacq X., Karpen G.H. (2017). Phase Separation Drives Heterochromatin Domain Formation. Nature.

[65] Mirny L.A., Imakaev M., Abdennur N. (2019). Two Major Mechanisms of Chromosome Organization. Curr. Opin. Cell Biol..

[66] Larson A.G., Elnatan D., Keenen M.M., Trnka M.J., Johnston J.B., Burlingame A.L., Agard D.A., Redding S., Narlikar G.J. (2017). Liquid Droplet Formation by HP1α Suggests a Role for Phase Separation in Heterochromatin. Nature.

[67] Holehouse A.S., Kragelund B.B. (2024). The Molecular Basis for Cellular Function of Intrinsically Disordered Protein Regions. Nat. Rev. Mol. Cell. Biol..

[68] Afanasyeva A., Bockwoldt M., Cooney C.R., Heiland I., Gossmann T.I. (2018). Human Long Intrinsically Disordered Protein Regions Are Frequent Targets of Positive Selection. Genome Res..

[69] Brown C.J., Johnson A.K., Dunker A.K., Daughdrill G.W. (2011). Evolution and Disorder. Curr. Opin. Struct. Biol..

[70] Molteni C., Forni D., Cagliani R., Mozzi A., Clerici M., Sironi M. (2023). Evolution of the Orthopoxvirus Core Genome. Virus Res..

[71] Mozzi A., Forni D., Cagliani R., Clerici M., Pozzoli U., Sironi M. (2020). Intrinsically Disordered Regions Are Abundant in Simplexvirus Proteomes and Display Signatures of Positive Selection. Virus Evol..

[72] Zarin T., Strome B., Nguyen Ba A.N., Alberti S., Forman-Kay J.D., Moses A.M. (2019). Proteome-Wide Signatures of Function in Highly Diverged Intrinsically Disordered Regions. eLife.

[73] Cagliani R., Forni D., Mozzi A., Fuchs R., Tussia-Cohen D., Arrigoni F., Pozzoli U., De Gioia L., Hagai T., Sironi M. (2024). Evolution of virus-like features and intrinsically disordered regions in retrotransposon-derived mammalian genes. Mol. Biol. Evol..

[74] Zhang R., Liu Y., Gao J. (2023). Phase Separation in Controlling Meiotic Chromosome Dynamics. Current Topics in Developmental Biology.

[75] Pontremoli C., Forni D., Cagliani R., Pozzoli U., Clerici M., Sironi M. (2018). Evolutionary Rates of Mammalian Telomere-Stability Genes Correlate with Karyotype Features and Female Germline Expression. Nucleic Acids Res..

[76] Pontremoli C., Forni D., Pozzoli U., Clerici M., Cagliani R., Sironi M. (2021). Kinetochore Proteins and Microtubule-destabilizing Factors Are Fast Evolving in Eutherian Mammals. Mol. Ecol..

[77] Henikoff S., Ahmad K., Malik H.S. (2001). The Centromere Paradox: Stable Inheritance with Rapidly Evolving DNA. Science.

[78] Wu C.-I., Yujun Xu E. (2003). Sexual Antagonism and X Inactivation—the SAXI Hypothesis. Trends Genet..

[79] Tenthorey J.L., Emerman M., Malik H.S. (2022). Evolutionary Landscapes of Host-Virus Arms Races. Annu. Rev. Immunol..

[80] Abdul F., Diman A., Baechler B., Ramakrishnan D., Kornyeyev D., Beran R.K., Fletcher S.P., Strubin M. (2022). Smc5/6 Silences Episomal Transcription by a Three-Step Function. Nat. Struct. Mol. Biol..

[81] Lee C.-P., Huang Y.-H., Lin S.-F., Chang Y., Chang Y.-H., Takada K., Chen M.-R. (2008). Epstein-Barr Virus BGLF4 Kinase Induces Disassembly of the Nuclear Lamina to Facilitate Virion Production. J. Virol..

[82] Takemoto A., Kimura K., Yanagisawa J., Yokoyama S., Hanaoka F. (2006). Negative Regulation of Condensin I by CK2-Mediated Phosphorylation. EMBO J..

[83] Kumar S., Stecher G., Suleski M., Hedges B. (2017). TimeTree: A Resource for Timelines, Timetrees, and Divergence Times. Mol. Biol. Evol..

