# Supplementary material for: Positive Selection Drives the Evolution of the Structural Maintenance of Chromosomes (SMC) Complexes
Source: Genes (Basel). 2024 Sep 3;15(9):1159. doi: 10.3390/genes15091159 (PMC11431564; doi:10.3390/genes15091159)

Supplementary Table S1. List of species analyzed in each SMC gene. A white square indicates that the species is missing for that gene.

Scientific name	**Common name**	**RAD21**	**RAD21L1**	**REC8**	**SMC1A**	**SMC1B**	**SMC3**	**STAG1**	**STAG2**	**STAG3**	**PDS5A**	**PDS5B**	**NCAPD2**	**NCAPD3**	**NCAPG**	**NCAPG2**	**NCAPH**	**NCAPH2**	**SMC2**	**SMC4**	**NSMCE1**	**NSMCE2**	**NSMCE3**	**NSMCE4A**	**EID3**	**SMC5**	**SMC6**
Aotus nancymaae	Nancy Ma's night monkey	■	■	■	■	■	■	■	■	■	□	■	■	■	■	■	■	■	■	■	■	■	■	■	□	■	■
Callithrix jacchus	Marmoset	■	■	■	■	■	■	■	■	■	■	■	■	■	■	■	■	■	■	■	■	■	■	■	■	■	■
Carlito syrichta/Tarsier syrichta	Philippine tarsier	■	■	■	■	■	■	■	■	■	■	■	■	■	■	■	■	■	■	■	■	□	■	■	□	■	■
Cebus capucinus	White-headed capuchin	■	■	■	■	■	■	■	■	■	□	□	■	■	■	■	■	■	■	■	■	■	■	■	□	■	■
Cercocebus atys	Sooty mangabey	■	■	■	■	■	■	■	■	■	■	■	■	■	■	□	■	■	■	■	■	■	□	■	□	■	■
Chlorocebus sabaeus	Green monkey	■	■	■	■	■	■	■	■	■	■	■	■	■	■	■	■	■	■	■	■	■	■	■	■	■	■
Colobus angolensis	Sclater's Angola colobus	■	■	■	■	■	■	■	■	■	■	■	■	■	■	■	■	■	■	■	■	■	□	■	□	■	■
Gorilla gorilla	Gorilla	■	■	■	■	■	■	■	■	■	■	■	■	■	■	■	■	■	■	■	■	■	■	■	■	■	■
Homo sapiens	Human	■	■	■	■	■	■	■	■	■	■	■	■	■	■	■	■	■	■	■	■	■	■	■	■	■	■
Macaca fascicularis	Crab-eating macaque	■	■	■	■	■	■	■	■	■	■	■	■	■	■	■	■	■	■	■	■	■	■	■	■	■	■
Macaca mulatta	Rhesus macaque	■	■	■	■	■	■	■	■	■	■	■	■	■	■	■	■	■	■	■	■	■	■	■	■	■	■
Microcebus murinus	Mouse lemur	■	■	■	■	■	■	■	■	■	■	■	■	■	■	■	■	■	■	□	■	■	■	■	□	■	■
Nomascus leucogenys	Gibbon	■	■	■	■	■	■	■	■	■	■	■	■	■	■	■	■	■	■	■	■	■	■	■	■	■	■
Otolemur garnettii	Bushbaby	■	■	■	■	■	■	■	■	■	■	■	■	■	■	■	■	■	■	■	■	■	■	■	■	■	■
Pan paniscus	Bonobo	■	■	■	□	■	■	■	■	■	■	■	□	■	■	■	■	■	■	■	■	■	■	■	□	■	■
Pan troglodytes	Chimpanzee	■	■	■	■	■	■	■	■	■	■	■	■	■	■	□	■	■	■	■	■	■	■	■	■	■	■
Papio hamadryas	Baboon	■	■	■	■	■	■	■	■	■	■	■	■	■	■	■	■	■	■	■	■	■	■	■	■	■	■
Pongo abelii	Orangutan	■	■	■	■	■	■	■	■	■	■	■	■	■	■	■	■	■	■	■	■	■	■	■	■	■	■
Rhinopithecus roxellana	Snub-nosed monkey	■	■	■	■	■	■	■	■	■	■	■	■	■	■	■	■	■	■	■	■	■	□	■	□	■	■
Saimiri boliviensis boliviensis	Squirrel monkey	■	■	■	■	■	■	■	■	■	■	■	■	■	■	■	■	■	■	■	■	■	■	■	■	■	■
Tupaia chinensis	Chinese tree shrew	■	■	■	■	■	■	■	■	■	■	■	■	■	■	■	■	■	■	■	■	■	■	■	■	■	■
Ochotona princeps	Pika	■	■	■	■	■	■	■	■	■	■	■	■	■	■	■	■	■	■	■	■	■	□	■	■	■	■
Oryctolagus cuniculus	Rabbit	■	■	■	■	■	■	■	■	■	■	■	■	■	■	■	■	■	■	■	■	■	■	■	□	■	■
Castor canadensis	North American beaver	■	■	■	■	□	■	■	■	■	■	■	■	■	■	■	■	■	■	■	■	■	■	■	□	■	■
Cavia porcellus	Guinea pig	■	■	■	■	■	■	■	■	■	■	■	■	■	■	■	■	■	■	■	■	■	■	■	■	■	■
Heterocephalus glaber	Naked mole-rat	■	■	■	■	■	■	■	■	■	■	■	■	■	■	■	■	■	■	■	■	■	■	■	■	■	■
Ictidomys tridecemlineatus	Squirrel	■	■	■	■	■	■	■	■	■	■	■	■	■	■	■	■	■	■	■	■	■	■	■	■	■	■
Jaculus jaculus	Lesser Egyptian jerboa	■	■	■	■	■	■	■	■	■	■	■	■	■	■	■	■	■	■	■	■	■	■	■	■	■	■
Marmota marmota	Alpin marmot	■	■	■	■	■	■	■	■	■	□	■	■	■	■	■	■	■	■	■	■	■	■	■	□	■	■
Mesocricetus auratus	Golden hamster	■	■	■	■	■	■	■	■	□	■	■	■	■	■	■	■	■	■	■	■	■	■	■	■	■	■
Microtus ochrogaster	Prairie vole	■	■	■	■	■	■	■	■	■	■	■	■	■	■	■	■	■	■	■	■	■	■	■	■	■	■
Mus musculus	Mouse	■	■	■	■	■	■	■	■	■	■	■	■	■	■	■	■	■	■	■	■	■	■	■	■	■	■
Peromyscus maniculatus	Deer mouse	■	■	■	■	■	■	■	■	■	■	■	■	■	■	■	■	■	■	■	■	■	■	■	□	■	■
Rattus norvegicus	Rat	■	■	■	■	■	■	■	■	■	■	■	■	■	■	■	■	■	■	■	■	■	■	■	■	■	■
Balaenoptera acutorostrata	Minke whale	■	■	■	■	■	■	■	■	■	■	■	■	■	■	■	■	■	■	■	■	■	■	■	■	■	■
Bos taurus	Cow	■	■	■	■	■	■	■	■	■	■	■	■	■	■	■	■	■	■	■	■	■	■	■	■	■	■
Camelus bactrianus	Bactrian camel	■	■	■	■	■	■	■	■	■	□	□	■	■	■	■	■	■	■	■	■	■	□	■	□	■	■
Orcinus orca	Killer Whale	■	■	■	■	■	■	■	■	■	■	■	■	■	■	■	■	■	■	□	□	■	■	■	□	■	■
Ovis aries	Sheep	■	■	■	■	■	■	■	■	■	■	■	■	■	■	■	■	■	■	■	■	■	■	■	■	■	■
Pantholops hodgsonii	Tibetan antelope	■	■	■	□	■	■	■	■	■	■	■	■	■	■	■	■	■	□	■	■	■	■	■	□	■	■
Physeter catodon	Sperm whale	■	■	■	■	■	■	■	■	■	■	■	■	■	■	■	■	■	■	■	■	■	■	■	■	■	■
Sus scrofa	Pig	■	■	■	■	■	■	■	■	■	■	■	■	■	■	■	■	■	■	■	■	■	□	■	■	■	■
Tursiops truncatus	Dolphin	■	■	■	■	■	■	■	■	■	■	■	■	■	■	■	■	■	■	■	■	■	■	■	■	■	■
Acinonyx jubatus	Cheetah	■	■	■	■	■	■	■	■	■	■	■	■	■	■	■	■	■	■	■	■	■	■	■	■	■	■
Ailuropoda melanoleuca	Giant panda	■	■	■	■	□	■	■	■	■	■	■	■	■	■	□	■	■	■	■	■	■	■	■	■	■	■
Canis lupus familiaris	Dog	■	■	■	■	■	■	■	■	■	■	■	■	■	■	■	■	■	■	■	■	■	■	■	■	■	■
Felis catus	Cat	■	■	■	■	■	■	■	■	■	■	■	■	■	■	□	■	■	■	■	□	■	■	■	■	■	■
Leptonychotes weddellii	Weddell seal	■	■	■	■	■	■	■	■	■	■	■	■	■	■	■	■	■	■	■	■	■	■	■	■	■	■
Mustela putorius furo	Ferret	■	■	■	■	■	■	■	■	■	■	■	■	■	■	■	■	■	■	■	■	■	■	■	■	■	■
Odobenus rosmarus divergens	Pacific walrus	■	■	■	■	■	■	■	■	■	■	■	■	■	■	■	■	■	■	■	■	■	■	■	■	■	■
Panthera pardus	Leopard	■	■	■	■	■	■	■	■	■	■	■	■	■	■	■	■	■	■	■	■	■	■	■	■	■	■
Eptesicus fuscus	Big brown bat	■	■	■	■	■	■	■	■	■	■	■	■	■	■	■	■	■	■	■	■	■	■	■	■	■	■
Pteropus vampyrus	Large flying fox	■	■	■	■	■	■	■	■	■	■	■	■	■	■	■	■	■	■	■	■	■	■	■	■	■	■
Rhinolophus sinicus	Chinese rufous horseshoe bat	■	■	■	■	■	■	■	■	■	■	■	■	■	■	■	■	■	■	■	□	■	■	■	□	■	■
Condylura cristata	Star-nosed mole	■	■	■	■	■	■	■	■	■	■	■	■	■	■	■	■	■	■	■	■	■	■	■	■	■	■
Erinaceus europaeus	European hedgehog	■	■	■	■	■	■	■	■	■	□	■	■	■	■	■	■	■	■	■	■	■	■	■	□	■	■
Sorex araneus	Common shrew	■	■	■	■	□	■	■	■	■	■	■	■	■	■	■	■	□	■	■	■	■	□	■	■	■	■
Ceratotherium simum simum	White rhinoceros	■	■	■	■	■	■	■	■	■	■	■	■	■	■	■	■	■	■	■	■	■	□	■	■	■	■
Equus caballus	Horse	■	■	■	■	■	■	■	■	■	■	■	■	■	■	■	■	■	■	■	■	■	■	■	■	■	■
Dasypus novemcinctus	Nine-banded armadillo	■	■	■	■	■	■	■	■	■	□	■	■	■	■	■	■	■	■	■	■	■	■	■	■	■	■
Echinops telfairi	Lesser hedgehog tenrec	■	■	■	■	■	■	■	■	■	■	□	■	■	■	■	■	■	■	■	■	■	□	■	■	■	■
Loxodonta africana	African elephant	■	■	■	■	■	■	■	■	■	■	■	■	■	■	■	■	■	■	■	■	■	■	■	■	■	■
Orycteropus afer	Aardvark	■	■	■	■	■	■	■	■	■	■	■	■	■	■	■	■	■	■	■	■	■	■	■	■	■	■

Supplementary Table S2. Likelihood ratio test statistics for models of variable selective pressure among sites (F3x4 and F61 codon frequency model) for Cohesin Complex genes.

Gene/ LRT model	n. of species	F3x4		F61		Positively selected sites[c]
		-2ΔlnL[a]	p value[b]	-2ΔlnL[a]	p value[b]	
**RAD21**	63					
M1 vs M2		0.00	1	0	1	
M7 vs M8		35.89	$1.61x10^{-08}$	25.59	$2.77x10^{-06}$	
**RAD21L***	63					
M1 vs M2		102.59	**$5.28x10^{-23}$**	92.78	**$7.13x10^{-21}$**	122,148,192,284,394,398,404, 411,477,433
M7 vs M8		113.97	**$1.79x10^{-25}$**	108.91	**$2.25x10^{-24}$**	
**REC8***	63					
M1 vs M2		51.13	**$7.89x10^{-12}$**	10.11	**0.0064**	152,168,191,199,253,264,269, 358,400,449,178,244
M7 vs M8		88.22	**$6.97x10^{-20}$**	50.28	**$1.21x10^{-11}$**	
**SMC1A**	61					
M1 vs M2		0	1	0	1	
M7 vs M8		0	1	0	1	
**SMC1B***	60					
M1 vs M2		37.77	**$6.29x10^{-09}$**	16.92	**0.00021**	6,18,251,491,877,1088
M7 vs M8		105.04	**$1.55x10^{-23}$**	55.29	**$9.85x10^{-13}$**	
**SMC3**	63					
M1 vs M2		0	1	0.34	0.84	
M7 vs M8		0	1	0	1	
**PDS5A**	57					
M1 vs M2		1.75	0.42	10.84	$4.43x10^{-03}$	
M7 vs M8		60.09	$8.95x10^{-14}$	70.90	$4.02x10^{-16}$	
**PDS5B**	60					
M1 vs M2		0	1	0.00	1	
M7 vs M8		41.99	$7.62x10^{-10}$	65.08	$7.38x10^{-15}$	
**STAG1**	63					
M1 vs M2		0	1	0	1	
M7 vs M8		47.27	$5.44x10^{-11}$	49.76	$1.57x10^{-11}$	
**STAG2**	63					
M1 vs M2		0	1	0	1	
M7 vs M8		36.52	$1.17x10^{-08}$	0	1	
**STAG3***	62					
M1 vs M2		27.39	**$1.13x10^{-06}$**	18.02	**0.00012**	24,83,86,764,862,1044,1089, 1154,1159,1197
M7 vs M8		79.88	**$4.51x10^{-18}$**	58.44	**$2.04x10^{-13}$**	

[a] Twice the difference of likelihood for the two models compared; [b] *p* value of rejecting the neutral models (M8a and M7) in favor of the positive selection model (M8); [c] positively selected sites detected by at least two methods among BEB, FEL, and FUBAR; * meiotic-specific cohesin.

Supplementary Table S3. Likelihood ratio test statistics for models of variable selective pressure among sites (F3x4 and F61 codon frequency model) for Condensin Complex genes.

**Gene/ LRT model**	**n. of species**	**F3x4**		**F61**		**Positively selected sites**[c]
		-2ΔlnL[a]	p value[b]	-2ΔlnL[a]	p value[b]	
**NCAPD2**	62					
M1 vs M2		11.19	0.0037	3.33	0.19	
M7 vs M8		104.18	$2.39x10^{-23}$	74.54	$6.51x10^{-17}$	
**NCAPD3**	63					
M1 vs M2		0	1	0	1	
M7 vs M8		71.47	$3.02x10^{-16}$	66.41	$3.80x10^{-15}$	
**NCAPG**	63					
M1 vs M2		46.98	**$6.29x10^{-11}$**	48.72	**$2.63x10^{-11}$**	36,37,84,616
M7 vs M8		90.97	**$1.76x10^{-20}$**	102.35	**$5.96x10^{-23}$**	
**NCAPG2**	59					
M1 vs M2		0	1	0	1	
M7 vs M8		48.45	$3.01x10^{-11}$	44.59	$2.084x10^{-10}$	
**NCAPH**	63					
M1 vs M2		0	1	0	1	
M7 vs M8		5.35	0.069	1.43	0.49	
**NCAPH2**	62					
M1 vs M2		0	1	0	1	
M7 vs M8		12.27	0.0022	42.82	$5.03x10^{-10}$	
**SMC2**	62					
M1 vs M2		0	1	0.13	0.94	
M7 vs M8		37.33	$7.83x10^{-09}$	36.32	$1.30x10^{-8}$	
**SMC4**	61					
M1 vs M2		11.76	0.0028	4.56	0.10	
M7 vs M8		114.13	$1.65x10^{-25}$	65.86	$5.00x10^{-15}$	

[a] Twice the difference of likelihood for the two models compared; [b] *p* value of rejecting the neutral models (M8a and M7) in favor of the positive selection model (M8); [c] positively selected sites detected by at least two methods among BEB, FEL, and FUBAR.

Supplementary Table S4. Likelihood ratio test statistics for models of variable selective pressure among sites (F3x4 and F61 codon frequency model) for SMC5/6 complex genes.

**Gene/ LRT model**	**n. of species**	**F3x4**		**F61**		**Positively selected sites[c]**
		-2ΔlnL[a]	p value[b]	-2ΔlnL[a]	p value[b]	
**SMC5**	63					
M1 vs M2		17.97	**0.000125**	7.91	**0.019**	797,38,542,33
M7 vs M8		61.40	**$4.65x10^{-14}$**	45.29	**$1.46x10^{-10}$**	
**SMC6**	63					
M1 vs M2		0	1	0	1	
M7 vs M8		48.42	$3.06x10^{-11}$	14.44	0.00073	
**NSMCE1**	60					
M1 vs M2		0	1	0	1	
M7 vs M8		0	1	0	1	
**NSMCE2**	63					
M1 vs M2		0	1	0	1	
M7 vs M8		8.02	0.018	4.57	0.10	
**NSMCE3**	54					
M1 vs M2		0	1	0	1	
M7 vs M8		0	1	0.27	0.87	
**NSMCE4A**	63					
M1 vs M2		33.96	**$4.22x10^{-08}$**	22.82	**$1.11x10^{-05}$**	14, 185
M7 vs M8		45.11	**$1.60x10^{-10}$**	35.79	**$1.69x10^{-08}$**	
**EID3**	46					
M1 vs M2		0	1	0	1	
M7 vs M8		14.24	$8.09x10^{-04}$	10.78	0.00456	

[a] Twice the difference of likelihood for the two models compared; [b] *p* value of rejecting the neutral models (M8a and M7) in favor of the positive selection model (M8); [c] positively selected sites detected by at least two methods among BEB, FEL, and FUBAR.

Supplementary Table S5. Analysis of dS for individual branches in the phylogenies of positively selected genes.

Gene	Total branches	Branches with dS>0.25	Branches with dS>0.5	Branches with dS>1
**Cohesin Complex**				
**RAD21L***	123	2	0	0
**REC8***	123	4	0	0
**SMC1B***	117	3	0	0
**STAG3***	121	1	0	0
**Condensin Complex**				
**NCAPG**	123	3	1	0
**SMC5/6 Complex**				
**SMC5**	123	2	0	0
**NSMCE4A**	123	2	2	0

Supplementary Table S6. Likelihood ratio test statistics for models of variable selective pressure among branches.

	Gene	-2ΔlnL	Degrees of freedom	*p*-value
**Cohesin complex**				
	RAD21	157.79	122	**0.0161**
	RAD21L	163.58	122	**0.00715**
	REC8	223.2	122	**6.28×10^{-08}**
	SMC1A	91.51	118	0.966
	SMC1B	455.46	116	**9.73×10^{-42}**
	SMC3	77.35	122	0.999
	PDS5A	194.41	110	**1.25×10^{-06}**
	PDS5B	243.99	116	**1.25×10^{-06}**
	STAG1	192.07	122	**5.32×10^{-05}**
	STAG2	169.13	122	**0.00308**
	STAG3	299.6	120	**2.35×10^{-17}**
**Condensin complex**				
	NCAPD2	194.08	121	**2.79×10^{-05}**
	NCAPD3	426.83	122	**1.95×10^{-35}**
	NCAPG	215.26	122	**3.95×10^{-07}**
	NCAPG2	220.16	114	**9.49×10^{-09}**
	NCAPH	223.74	122	**5.53×10^{-08}**
	NCAPH2	137.62	120	0.129
	SMC2	298.82	120	**2.97×10^{-17}**
	SMC4	282.92	118	**1.43×10^{-15}**
**SMC5/6 complex**				
	NSMCE1	161.69	116	**0.00327**
	NSMCE2	235.89	120	**1.44×10^{-09}**
	NSMCE3	157.00	104	**0.000612**
	NSMCE4A	287.30	122	**2.31×10^{-15}**
	EID3	169	88	**5.61×10^{-31}**
	SMC5	368.71	122	**1.33×10^{-26}**
	SMC6	388.60	122	**1.46×10^{-29}**

Note: ΔLnL is the difference of the natural logs of the maximum likelihood of a null model that assumes all branches to have the same dN/dS with a model that allows each branch to have its own dN/dS.

Supplementary Figure S1. dN/dS variability among mammalian species. Representative phylogenetic tree of mammalian species analyzed herein. Colored dots represent tree branches showing dN/dS value >1 estimated by the MFR model for that specific group. See the legend for color details.

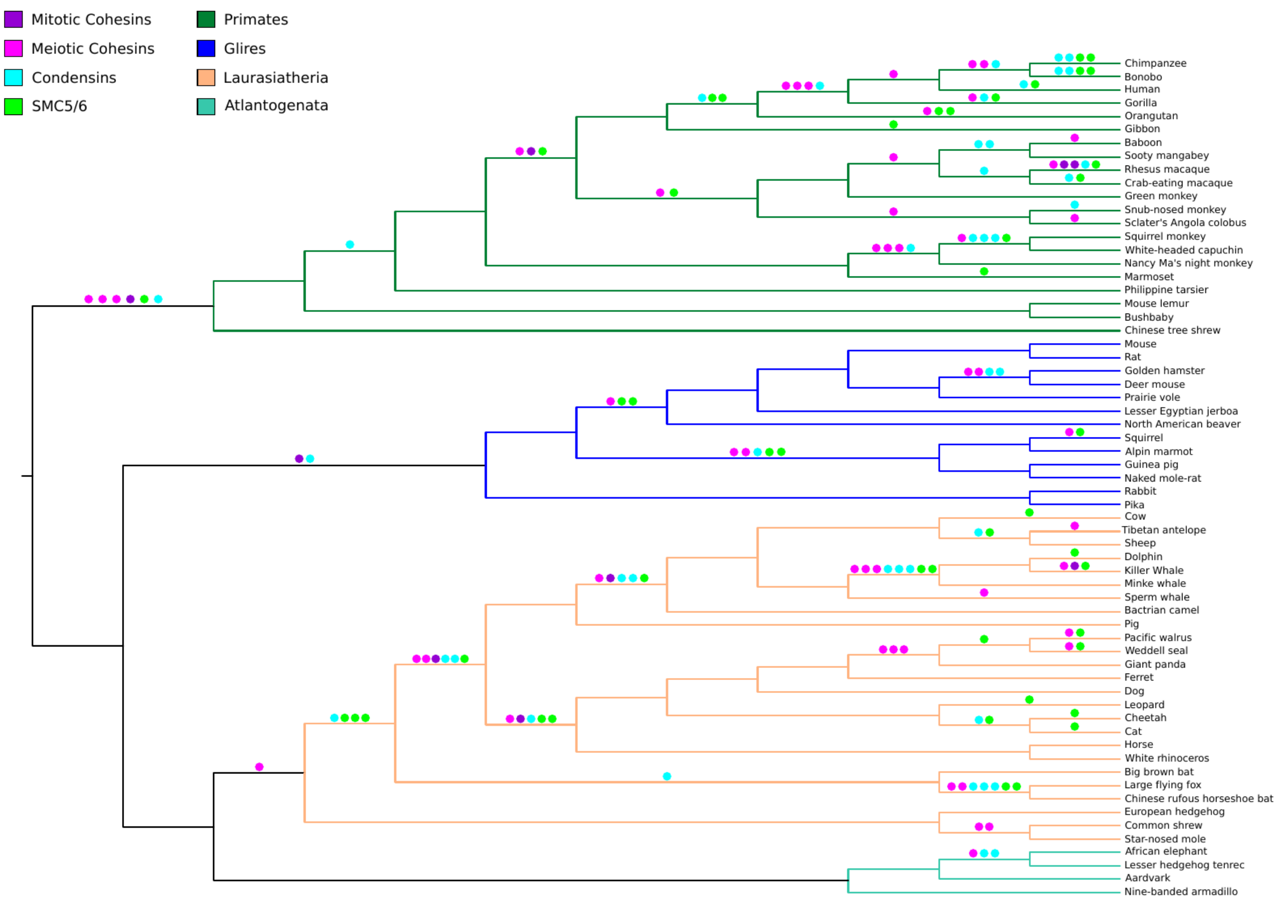

Supplement: Supplementary file 1 [file genes-15-01159-s001.zip › genes-3160731-supplementary.pdf]
